# Supplementary material for: Comparison of the Predictive Accuracy of Intraocular Lens Power Calculations after Phototherapeutic Keratectomy in Granular Corneal Dystrophy Type 2
Source: J Clin Med. 2023 Jan 11;12(2):584. doi: 10.3390/jcm12020584 (PMC9861484; doi:10.3390/jcm12020584)
Supplement: Supplementary file 1 [file jcm-12-00584-s001.zip › jcm-1926903-supplementary.pdf]

Table S1. Additional photorefractive keratectomy (PRK) calculation table after phototherapeutic keratectomy (PTK).

	PTK ablation depth		Add PRK (D)	PRK ablation depth (μm)	Total CAD (μm)
	CAD (μm)	PAD (μm)			
6.0 mm Treatment zone	50	57.5	-0.5	7.5	57.5
Calculation	5	5.7	-0.05	0.68	5.68
	10	11.4	-0.1	1.35	11.35
	15	17.1	-0.16	2.16	17.16
	20	22.8	-0.21	2.83	22.83
	25	28.5	-0.26	3.50	28.50
	30	34.2	-0.31	4.17	34.17
	35	39.8	-0.36	4.84	39.84
	40	45.5	-0.41	5.51	45.51
	45	51.2	-0.46	6.18	51.18
	50	56.9	-0.51	6.84	56.84
	55	62.6	-0.57	7.64	62.64
	60	68.3	-0.62	8.31	68.31
	65	74.0	-0.67	8.97	73.97
	70	79.7	-0.72	9.63	79.63

CAD: Central ablation depth, PAD: Peripheral ablation depth, PTK: Phototherapeutic keratectomy, PRK: Photorefractive keratectomy, D: diopter
